# Downregulation of circ_0132266 in chronic lymphocytic leukemia promoted cell viability through miR-337-3p/PML axis

**DOI:** 10.18632/aging.101997

**Published:** 2019-06-01

**Authors:** Wei Wu, Zijuan Wu, Yi Xia, Shuchao Qin, Yue Li, Jiazhu Wu, Jinhua Liang, Li Wang, Huayuan Zhu, Lei Fan, Jianxin Fu, Wei Xu, Hui Jin, Jianyong Li

**Affiliations:** 1 Department of Hematology, The First Affiliated Hospital of Nanjing Medical University, Jiangsu Province Hospital, Nanjing, 210029, China; 2 Key Laboratory of Hematology of Nanjing Medical University, Nanjing, 210029, China; 3 Collaborative Innovation Center for Cancer Personalized Medicine, Nanjing, 210029, China

**Keywords:** circular RNA, circRNAs, chronic lymphocytic leukemia, CLL, miR-337-3p, PML, ceRNA

## Abstract

Circular RNAs (circRNAs) have recently been reported to play crucial roles in various regulatory processes and involved in cancer onset and progression. However, the potential mechanism of circRNAs in chronic lymphocytic leukemia (CLL) remains largely unknown. Here, we observed hsa_circ_0132266 (circ_0132266), a circRNA significantly decreased in the peripheral blood mononuclear cells (PBMCs) of CLL patients compared with healthy donors, could act as an endogenous sponge of hsa-miR-337-3p (miR-337-3p) and regulate its activity, which resulted in a downstream change of target-gene PML and a consequent influence on cell viability. Taken together, our data indicated the regulatory mechanism of circ_0132266 in CLL progression through circ_0132266/miR-337-3p/PML axis, suggesting that it may serve as a biomarker as well as an exploitable therapeutic target for CLL.

## INTRODUCTION

Chronic lymphocytic leukemia (CLL), a heterogeneous disease characterized by the monoclonal expansion of B cells, is the commonest leukemia in the western hemisphere [1, 2]. However, the pathogenesis mechanism of CLL remains elusive. MicroRNAs (miRNAs) are a large class of non-coding RNAs (ncRNAs) involved in tumorigenesis and have been reported to exert effects in the progression of multiple diseases [3–5]. Several current studies have shed lights on the aberrantly expressed miRNAs in CLL and demonstrated their potential roles as novel biomarkers and therapeutic targets [6, 7]. For example, the upregulated miR-92a-3p in CLL patients predicts a favorable prognostic outcome [8]. miR-125a-5p and miR-34a-5p are considered as promising indicators of CLL Richter syndrome transformation [9]. miR-34a is revealed to participate in B-cell receptor (BCR) signaling and thus affects CLL patients’ treatment [10].

miR-337-3p which has been detected to be downregulated in clear cell renal cell carcinoma [11], gastric cancer [12] and hepatocellular carcinoma [13, 14], could function as a suppressive factor in cell proliferation, tumor growth and metastasis through directly regulating target genes. Contrary to these previous reports, in our research, we accidentally found that miR-337-3p exhibited obviously high expression in peripheral blood mononuclear cell (PBMCs) of CLL patients compared with normal controls. The current results inspired us to further explore its regulatory mechanism.

Circular RNAs (circRNAs), widely expressed in human transcriptome, have been disclosed to function as a competitive endogenous RNA (ceRNA) via sponging miRNAs and further responsible for the expression of mRNA and the occurrence and development of diseases [15, 16]. Herein, we identified a CLL-related circRNA, circ_0132266, with an obviously low expression level and was negatively associated with miR-337-3p. Our research, for the first time, uncovered the functions of circ_0132266, which could compete for miR-337-3p and further influence the expression abundance of promyelocytic leukemia protein (PML) and thus promote CLL progression. Our results may provide a novel diagnostic and therapeutic candidate for CLL.

## RESULTS

### miR-337-3p upregulated in CLL, promoted cell proliferation and inhibited cell apoptosis

**Figure 1:**
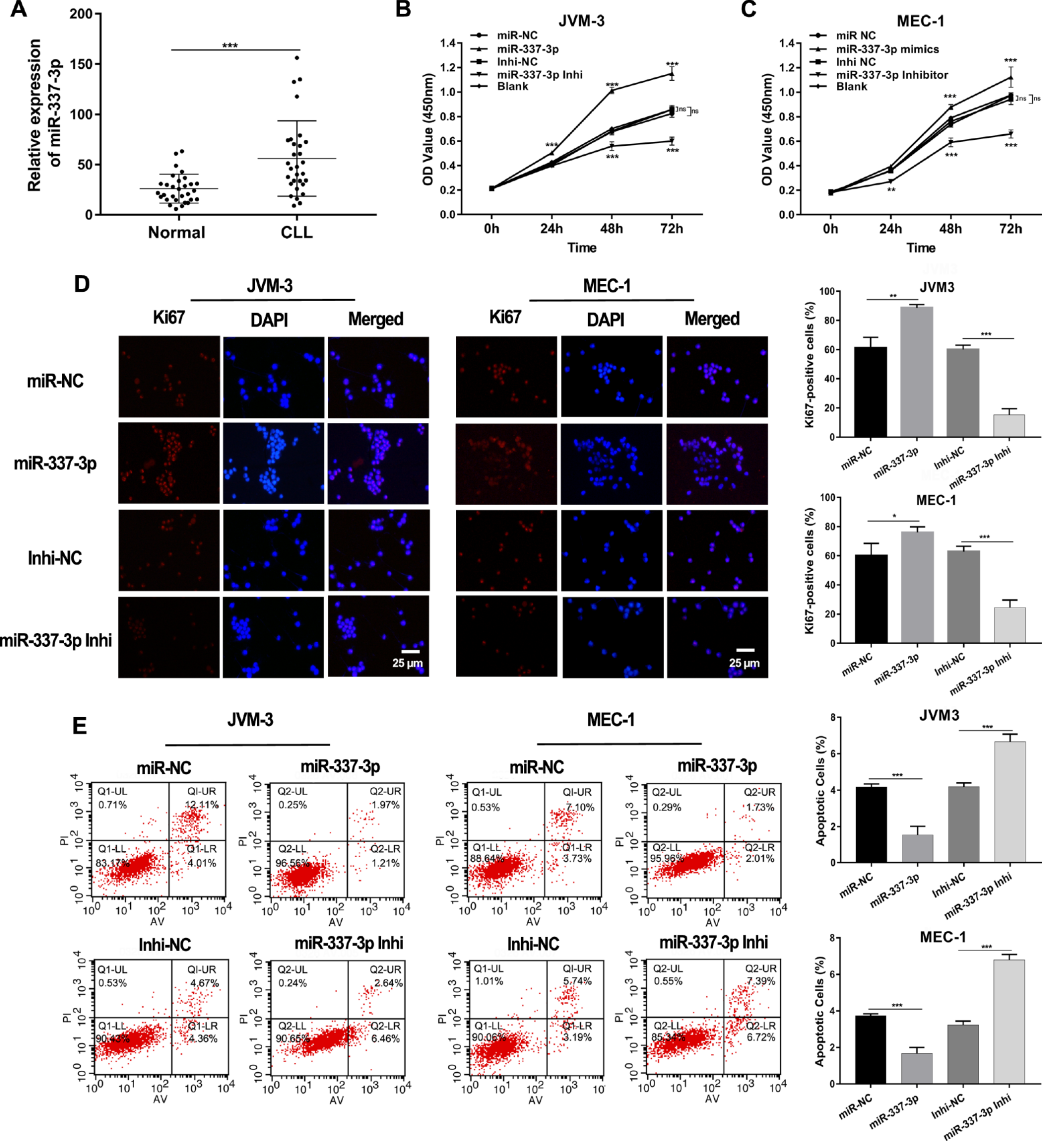
**Expression and functions of miR-337-3p in CLL.** (**A**) Relative expression levels of miR-337-3p in PBMC samples from thirty treatment-naïve CLL patients and thirty normal donors. (**B**, **C**) CCK8 assay was performed to assess the influence of miR-337-3p on CLL cells proliferation. (**D**) IF assay analyzed the proliferative viability of JVM-3 and MEC-1 after transfected with miR-337-3p mimics and inhibitor and ki67-positive cells rate were calculated. (**E**) Cell apoptosis was detected by FCM to verify the effects of miR-337-3p and the percentage of apoptotic cells was quantified. Three biological replicates were performed per condition and mean values ± SD are displayed (*P < 0.05; **P < 0.01; ***P < 0.001; ns, not significant).

Our previous studies occasionally noted that miR-337-3p was upregulated in CLL patients compared with healthy individuals. To further explore the expression pattern of miR-337-3p, we collected PBMC samples from thirty newly diagnosed CLL patients and thirty healthy volunteers. An increased expression of miR-337-3p was validated through qRT-PCR analysis, confirming the observations of our previous study (Figure 1A).

Subsequent cell proliferation and apoptosis assays were performed to dissect functions of miR-337-3p in CLL. Two CLL cell lines (MEC-1 and JVM-3) were used in our research. The growth curves of cells were detected via CCK8 assay. The results showed that ectopic expression of miR-337-3p promoted cell growth and this could be rescued by inhibition of miR-337-3p (Figure 1B, 1C, Supplementary Figure 1). The proliferation potential detected by immunofluorescence (IF) also assured the functions induced by miR-337-3p (Figure 1D). Flow cytometry examined the cell apoptosis level (Figure 1E), indicating that miR-337-3p could inhibit cell apoptosis and the quantification of apoptotic cell was shown in Figure 1G.

### Annotation for target genes of miR-337-3p

**Figure 2:**
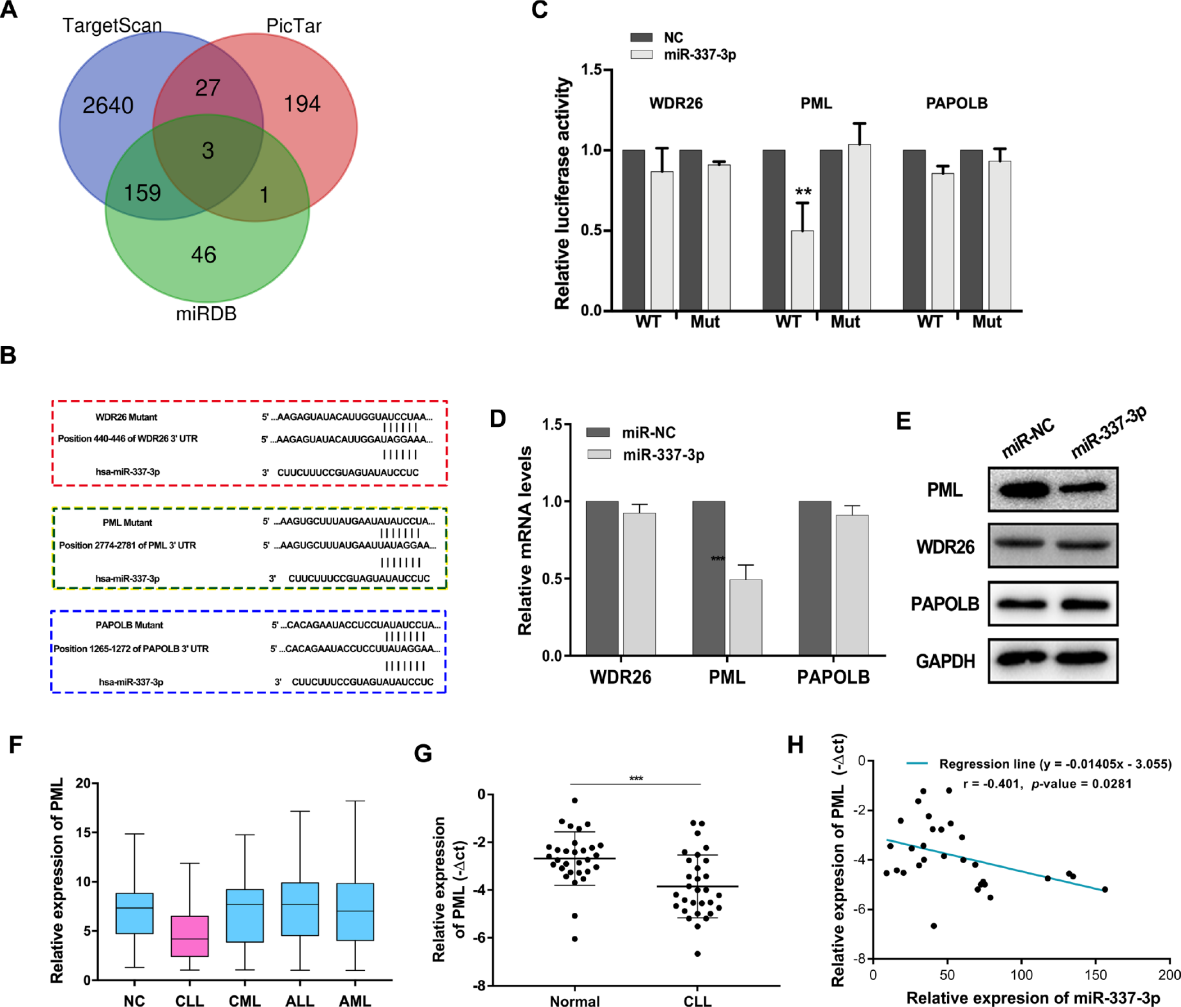
**PML is a target gene of miR-337-3p.** (**A**) Target genes were screened through overlapping websites of Targetscan (http://www.targetscan.org/vert_71/), miRDB (http://mirdb.org/) and Pictar (https://pictar.mdc-berlin.de/). (**B**) Potential binding sites between miR-337-3p and candidate genes. (**C**) Dual-luciferase reporter assay to verify predicted bindings. (**D**, **E**) The change of mRNAs and proteins expression after transfection with miR-337-3p mimics. (**F**) PML relative expression in leukemia and patients from TCGA database. (**G**) PML relative expression in CLL and normal patients PBMCs detected by qRT-PCR (30 CLL patients vs 30 healthy volunteers). (**H**) Correlation analysis between miR-337-3p and PML in CLL PBMCs.

To address how miR-337-3p functioned, we used bioinformatic analysis to forecast the target genes directly modulated by miR-337-3p. Three genes, WD repeat domain 26 (WDR26), promyelocytic leukemia protein (PML), poly(A) polymerase beta (PAPOLB) were preliminarily screened through overlapping the prediction results of miRNA recognition elements by Targetscan, miRDB and Pictar (Figure 2A). And the postulated binding sites between miR-337-3p and the candidate genes were shown in Figure 2B. Luciferase reporter vectors containing the wild type (WT) or mutant genes 3′UTR and miR-337-3p mimics were co-transfected into HEK-293T cells. Intriguingly, the luciferase activity of wild type PML 3′UTR evidently decreased in miR-337-3p mimics group while the activity of WDR26 and PAPOLB exhibited no difference (Figure 2C). Simultaneously, we detected the mRNA expression levels of WDR26, PML, PAPOLB and noted that transfection of miR-337-3p mimics downregulated the expression of PML (Figure 2D), these results were confirmed by western blot (Figure 2E). PML, originally identified to be involved in the rise of PML–RARα and RARα–PML fusion proteins, is a well-known tumor suppressor by regulating cell apoptosis and cell cycle [17–20]. Through TCGA database, we noticed that PML was obviously downregulated in CLL patients (Figure 2F). Our results further verified the low expression of PML and the negative correlation between PML and miR-337-3p expression detected by qRT-PCR in a newly diagnosed CLL cohort (Figure 2G, 2H). These results collectively indicated that PML was a direct target of miR-337-3p and miR-337-3p exerted functions of promoting cell proliferation and protecting cells from apoptosis.

### Circ_0132266, identified as a novel downregulated circRNA in CLL, was a potential modulator of miR-337-3p

CircRNAs, a novel type of ncRNAs, have been proposed to regulate mRNAs expression and ultimately participate in the progression of diseases through sponging miRNAs [15, 21]. Since miR-337-3p was upregulated in CLL whereas previous reports have elucidated that downregulated miR-337-3p in solid tumors could promote tumor growth, invasion, and metastasis [12, 13], it inspired our interest in exploring whether miR-337-3p was modulated by specific circRNA(s).

**Figure 3:**
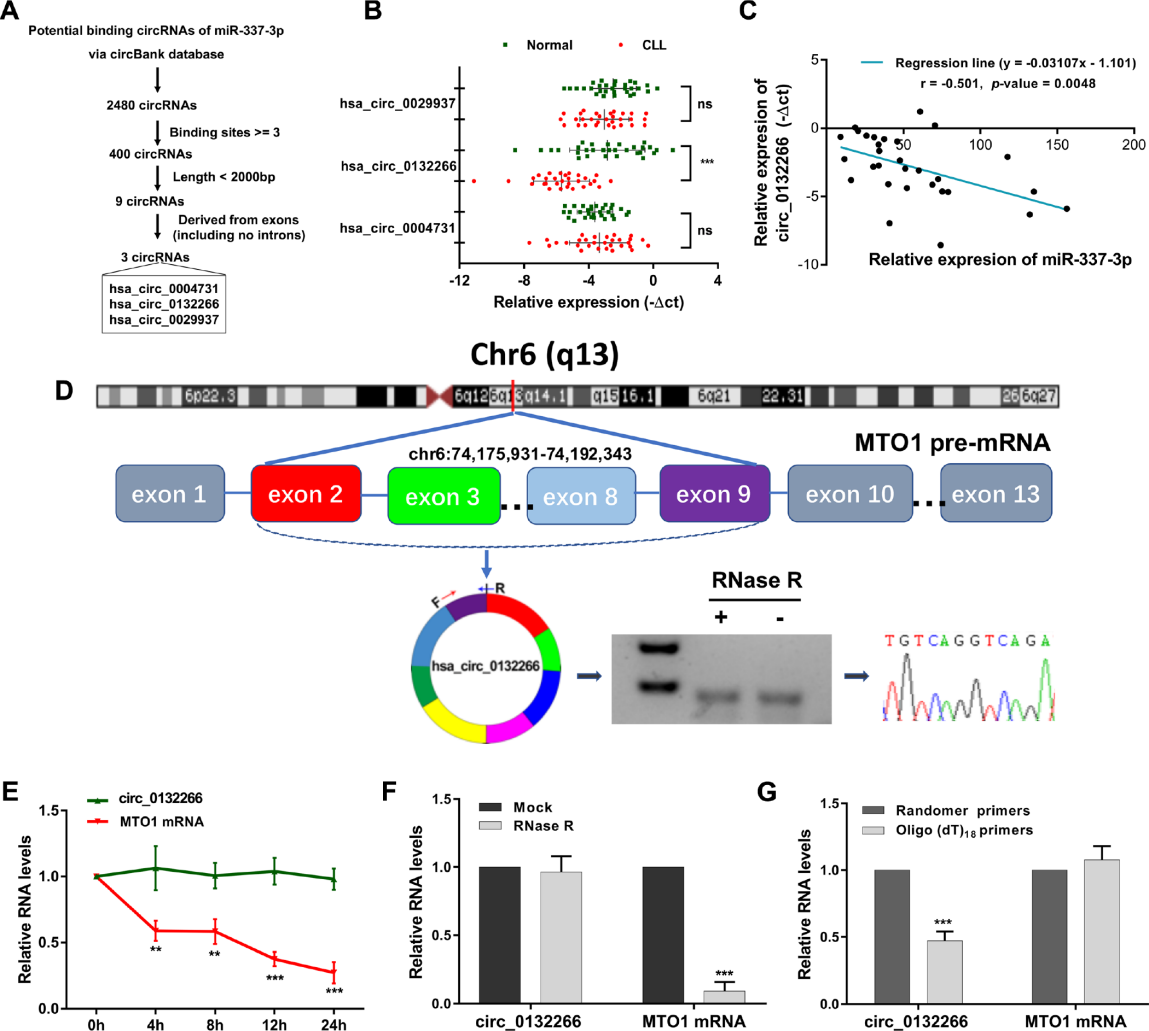
**Characteristics of circ_0132266 in CLL patients and cell lines.** (**A**) The schematic showed the screening rules of circRNAs that might regulate miR-337-3p. (**B**) Relative expression of the three candidate circRNAs in CLL patients and healthy individuals (n=30). (**C**) Expression correlation between miR-337-3p and circ_0132266 in CLL PBMCs. (**D**) The genomic loci of the MTO1 gene and circ_0132266. The expression of circ_0132266 was detected by qRT-PCR and was validated by Sanger sequencing. The relative expression of circ_0132266 and MTO1 mRNA after treatment of (**E**) actinomycin D and (**F**) RNase R. (**G**) qRT-PCR detected the levels of circ_0132266 and MTO1 mRNA after reverse transcribed with random primers and oligo (dt)_18_ primers.

To determine the circRNA(s) responsible for the regulation of miR-337-3p, we screened circBank database (http://www.circbank.cn) through a detailed screening system and finally screened out three most promising candidates, hsa_circ_0004731, hsa_circ_0132266 (circ_0132266), hsa_circ_0029937 (Figure 3A). The abundance of the three circRNAs was then examined in CLL and normal patients. Circ_0132266 was remarkably decreased in CLL patients while the other two had no difference (Figure 3B). Meanwhile, the expression of miR-337-3p was found to be negatively associated with circ_0132266 (Figure 3C).

Circ_0132266 was derived from exon2-9 of the MTO1 gene on chromosome 6. The spanning junction primers were designed and expected size of product was amplified and verified by Sanger sequencing (Figure 3D). RNase R and actinomycin D treatment manifested the stability of circ_0132266 (Figure 3E, 3F). qRT-PCR analysis following the use of oligo (dt)_18_ primers and random primers in reverse transcription illustrated that circ_0132266 was a closed loop structure (Figure 3G). The results suggested the characteristics of circ_0132266 which was downregulated in CLL.

### Circ_0132266 was validated to be miR-337-3p sponge

**Figure 4:**
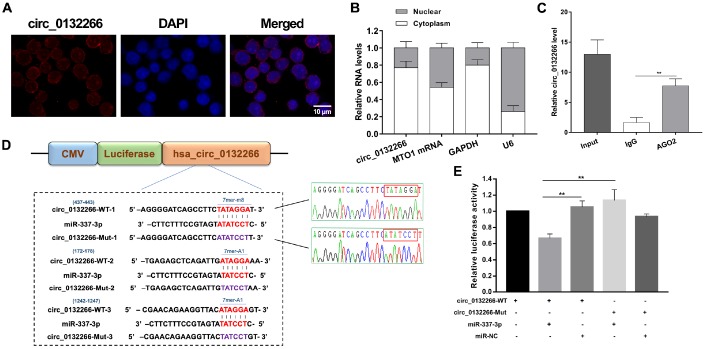
**Circ_0132266 serves as a sponge for miR-337-3p.** (**A**, **B**) FISH and nuclear and cytoplasmic fraction experiments were performed to detect the location of circ_0132266. (**C**) RIP assay performed with AGO2 antibody to assure the ability of circ_0132266 to serve as miRNA sponge. (**D**) Schematic showed the predicted binding sites between circ_0132266 and miR-337-3p and Sanger sequencing confirmed the accuracy of conducted vectors which will be used in the dual-luciferase assay. (**E**) The binding between circ_0132266 and miR-337-3p was verified through dual-luciferase activity assay.

Given to the expression correlation between circ_0132266 and miR-337-3p, we hypothesized that circ_0132266 functioned as miR-337-3p sponge. FISH assay and nuclear and cytoplasmic fraction experiments showed circ_0132266 was abundant in cytoplasm (Figure 4A, 4B). RIP assay showed the enrichment of circ_0132266 by AGO2 antibodies compared with IgG (Figure 4C). To further assure the binding of predicted putative binding sites of circ_0132266 and miR-337-3p (Figure 4D), one of the most potential bindings with highest score was detected through dual luciferase assay. As expected, there was a significant decrease of the luciferase activity mediated by wild-type circ_0132266 sequence when co-transfecting miR-337-3p mimics and Sanger sequencing assured the binding sites of conducted plasmids (Figure 4D, 4E).

Taken together, our data revealed that circ_0132266 possessed miRNA-binding sites and acted as a sponge of miR-337-3p.

### Circ_013226 was positively correlated with PML and affecting cell growth by sponging miR-337-3p

**Figure 5:**
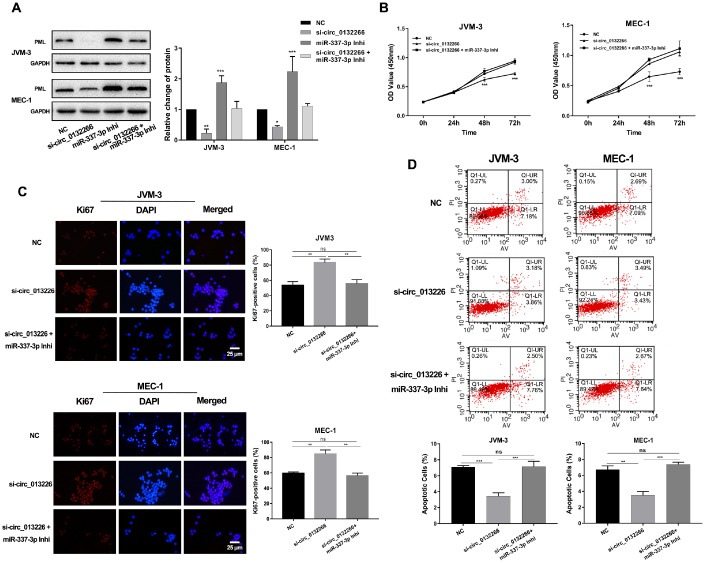
**Circ_013226 exerts functions through miR-337-3p/PML axis.** (**A**) The change of PML protein level after transfection with si-circ_0132266 and/or miR-337-3p inhibitor detected by western blot. (**B**, **C**) Cell proliferation potentials and ability were detected by CCK8 and IF assay. The statistical percentage of Ki67-positive cells was calculated. (**D**) FCM showed the circ_0132266 induced cell apoptosis ability and the rescue effects of miR-337-3p. Apoptotic rates were calculated and analyzed.

Since circ-013226 has a low expression in CLL patients, we decided to further investigate its cancer suppress effects through miR-337-3p/ PML axis. Three small interfering RNAs (siRNAs) against back-splice sequence were designed then the interfering efficiency was carried out via qRT-PCR and si-circ_0132266-1 was finally selected for our later experiments (Supplementary Figure 2A). Western blot analysis verified the results of qRT-PCR and indicated that the protein level of PML was decreased after RNA interfering (RNAi) of circ_0132266, which could be reversed by miR-337-3p inhibitor (Figure 5A). CCK8 assay and immunofluorescent (IF) staining of ki67 showed that circ_0132266 could inhibit the proliferation of CLL cells and this result could be alleviated by miR-337-3p inhibitor (Figure 5B, 5C, Supplementary Figure 2B, 2C). Apoptosis assay performed by FCM demonstrated that RNAi of circ_0132266 intensively suppressed cell apoptosis (Figure 5D).

**Figure 6:**
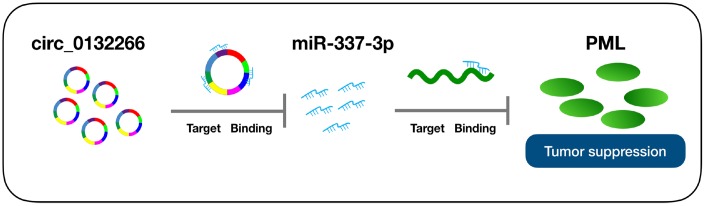
**Schematic representation of the proposed mechanism of circ_0132266 in CLL.** circ_0132266 acts as a miR-337-3p sponge to regulate the miR-337-3p/PML pathway. Decreased circ_0132266 in CLL leads to the upregulation of miR-337-3p, which eliminates the suppression effects of PML in CLL progression.

To sum up, our results implied that that circ_0132266 may be a tumor suppressive factor in CLL cells that functions as a decoy of miR-337-3p to regulate PML expression (Figure 6).

## DISCUSSION

Though CLL is the commonest leukemia in western countries and shows an increasing tendency in China, its pathogenesis has not yet been clear. It is therefore in urgent need of exploring the molecular mechanism of CLL.

In the present study, we noted an obviously upregulated miR-337-3p which has been reported to be aberrantly regulated in solid tumors and possessed tumor suppressing activity [11, 13, 22]. It is becoming increasingly evident that circRNAs as emerging ncRNAs have been uncovered preferential roles in mediating miRNAs and contributing to disease progression [23–25]. Therefore, we speculated a modulation mechanism of miR-337-3p mediated by circRNAs. Through a screening system of the genomic region of miR-337-3p, we identified and characterized the existence of a CLL-related circRNA, circ_0132266, and defined its expression profile and functions. Our results revealed a significant negative correlation between the expression levels of miR-337-3p and circ_0132266. And the dual luciferase reporter assay facilitated to support our propose. It is a reasonable speculation that circ_0132266 could accelerated CLL cells apoptosis and impaired proliferation through miR-337-3p/PML axis. Our research model not only opens a new avenue for understanding the regulatory mechanism underlying miR-337-3p but also highlights the possibility of considering circ_0132266 as a potential therapeutic target.

However, limitations cannot not be neglected in our research. Our future work will focus on the following explorations: Firstly, validate the expression of miR-337-3p in a larger cohort of CLL patients. Retrospective analysis is needed to be carried out by following up patients, aimed at finding out the relationship between miR-337-3p and the diagnosis, treatment and prognosis of CLL patients. Considering that our screen system might rule out circRNAs that participated in the process, the next step would be to add the whole transcriptome which will enable the full disclosure of regulatory mechanism of miR-337-3p. A body of evidence indicated that PML is a tumor suppressor and promising therapeutic targets, might be associated with chemo-resistant as well [26–28]. Nextly, gain and loss function assay of PML, a target gene presumably involved in CLL progression, will be performed to further elucidate its functions in order to provide a novel strategy to treat CLL.

Mounting evidence has shown that circRNAs sponging miRNAs might regulate gene transcription and play predominately roles in tumorigenesis. Our findings may enrich the knowledge of circ_0132266 induced mechanism leading to altered miR-337-3p and thus influence the proto-oncogene PML. In summary, the present study defines a novel mechanism of CLL progression and have important therapeutic implications for better treatment in the way of clinical therapy. The prospect of developing miRNA and circRNA-based interventions is undoubtedly an exciting one in the future.

## MATERIALS AND METHODS

### Patient samples

For the expression study, samples from thirty treatment-naïve patients diagnosed with CLL and thirty healthy volunteers were obtained after informed consent at the First Affiliated Hospital of Nanjing Medical University, Jiangsu Province Hospital. PBMCs were isolated by density gradient centrifugation with Lymphoprep™ (Stemcell technologies, Canada). The study was approved by the clinical investigation ethical committee from Jiangsu Province Hospital.

### Cell culture and transfection

MEC-1 purchased from Cobioer (Nanjing, China) and JVM-3 purchased from X-Y Biotechnology (Shanghai, China) were cultured in RPMI-1640 medium (Gibco®) supplemented with 10% fetal bovine serum (FBS, Gibco, Grand Island, USA), 100 μg/mL penicillin/streptomycin (PS, Gibco, Grand Island, USA) at 37 °C in a humidified atmosphere (5% CO_2_). Lipofectamine Stem reagent (Invitrogen, Carlsbad, USA) according to manufacturer’s instructions was used for cell transfection. HEK-293T obtained from X-Y Biotechnology (Shanghai, China) was maintained at 37°C with 5% CO_2_ in DMEM Medium, supplemented with PS, 10% FBS and was transfected using Lipofectamine 2000 (Invitrogen, Carlsbad, USA) according to manufacturer’s protocol.

### RNA isolation and quantitative RT-PCR

Total RNA was isolated using the Trizol method (Ambion, USA) and then transcribed reversed by TaqMan reverse transcription kit and amplified using the SYBR Green Master Mix (Applied Biosystems). The primers of mRNA and circRNAs were listed in Supplementary Table 1. miRNA was reversed and amplified by miR 1^st^ strand cDNA Synthesis Kit and miRNA Universal SYBR qPCR Master Mix (Vazyme, China), using specific miR-377 primers GTCGTATCCAGTGCAGGGTCCGAGGTATTCGCACTGGATACGACGAAGAA (RT) and CGCGCTCCTATATGATGCCT (forward). The U6 small nuclear RNA primer: 5′-CTCGCTTCGGCAGCACA-3′ (forward) and 5′-AACGCTTCACGAATTTGCGT-3′ (reverse) were used as an internal control.

### Cell counting kit-8 (CCK8) assay

Cell Counting Kit-8 (Beyotime, Shanghai, China) was used to detect cell proliferative ability. Cells were plated on 96-well plates and 10 μl/ well CCK8 solution were added at pointed time. Then the absorbance at 450 nm was measured after 2 h incubation.

### Cell apoptosis assay

Cells were collected and stained by propidium iodine/Annexin V-FITC staining (BD Biosciences) and then detected by flow cytometry (FCM) to analyze the apoptosis. For each cell line triplicates were performed.

### Immunofluorescence (IF) and RNA fluorescence in situ hybridization (FISH)

MEC-1 and JVM-3 were plated on slides in 48-well plates and incubated at 60°C for IF and RNA-FISH analysis. After fixation and permeabilization, cells were incubated overnight at 4°C with primary diluted antibody anti-Ki67 (#ab15580, Abcam). The secondary antibody Alexa Fluor® 594 - Conjugated Goat anti-Rabbit IgG (H+L) was purchased from ZSGB-BIO, China. Fluorescence were observed using Axio Scope.A1 (Zeiss). FISH assay was performed using Fluorescent In Situ Hybridization Kit (RiboBio, Guangzhou, China) according to the manufacturer’s guidelines. And Cy3-labeled probes from RiboBio, Guangzhou, China were measured by visualized with a confocal microscopy.

### Western blotting

Western blotting was performed using anti-Bax (# ab32503, Abcam), anti-Bcl-2 (# ab32124, Abcam), anti-PML (# ab179466, Abcam), anti-WDR26 (# ab 85961, Abcam), anti-PAPOLB (# ab163420, Abcam), and anti-GAPDH (# ab8245, Abcam) antibodies. Secondary antibodies used in the experiments were from Santa Cruz (Santa Cruz, CA, USA). And the blots were visualized using an enhanced chemiluminescence kit (Pierce, Waltham, USA).

### RNA immunoprecipitation (RIP)

RIP experiments were performed using the Magna RIP RNA-Binding Protein Immunoprecipitation Kit (Millipore, Bedford, MA) according to the manufacturer’s instructions. The probes were designed and synthesized by Sangon Biotech. The abundance of circ_0132266 and miR-337-3p was detected by qRT–PCR assay.

### Dual luciferase reporter assay

The interactions between circ_0132266, miR-337-3p and PML were measured using pMIR-REPORT^TM^ system (Applied Biosystems, USA). The circ_0132266 and mRNA 3′ UTR sequences containing wild-type (WT) or mutant (Mut) miR-337-3p binding sites were synthesized and respectively inserted into pMIR-REPORT vectors and then co-transfected with miR-337-3p mimics or control mimics using Lipofectamine 2000. Dual luciferase activities were tested by the dual-luciferase reporter assay kit (Promega, Madison, WI, USA) 48 h later.

### Statistical analysis

GraphPad Prism 7 (GraphPad Software Inc., La Jolla, CA) was used to analyze experimental data. The expression RNA levels were performed using student’s t test and a *p* value less than 0.05 was considered to be statistically significant.

## CONCLUSIONS

In summary, the findings of the present study define a novel mechanism of CLL deregulation and describe a new role of miR-337-3p as a carcinogen in CLL. And circ_0132266 which was demonstrated to be downregulated in CLL could sponge miR-337-3p and be involved in CLL progression through miR-337-3p/PML axis. Accordingly, our findings highlighted that circ_0132266 might have potential values for understanding the complicated molecular mechanisms of miR-337-3p in CLL and providing a new strategy for future CLL therapy.

## Supplementary Material

Supplementary Table and Figures

## References

[1] Hallek M. Chronic lymphocytic leukemia: 2017 update on diagnosis, risk stratification, and treatment. Am J Hematol. 2017; 92:946–65. 10.1002/ajh.24826 28782884

[2] Chiorazzi N, Rai KR, Ferrarini M. Chronic lymphocytic leukemia. N Engl J Med. 2005; 352:804–15. 10.1056/NEJMra041720 15728813

[3] Ambros V. The functions of animal microRNAs. Nature. 2004; 431:350–55. 10.1038/nature02871 15372042

[4] Halvorsen AR, Helland Å, Gromov P, Wielenga VT, Talman MM, Brunner N, Sandhu V, Børresen-Dale AL, Gromova I, Haakensen VD. Profiling of microRNAs in tumor interstitial fluid of breast tumors - a novel resource to identify biomarkers for prognostic classification and detection of cancer. Mol Oncol. 2017; 11:220–34. 10.1002/1878-0261.12025 28145100 PMC5527454

[5] Powers JT, Tsanov KM, Pearson DS, Roels F, Spina CS, Ebright R, Seligson M, de Soysa Y, Cahan P, Theißen J, Tu HC, Han A, Kurek KC, et al. Multiple mechanisms disrupt the let-7 microRNA family in neuroblastoma. Nature. 2016; 535:246–51. 10.1038/nature18632 27383785 PMC4947006

[6] Musilova K, Mraz M. MicroRNAs in B-cell lymphomas: how a complex biology gets more complex. Leukemia. 2015; 29:1004–17. 10.1038/leu.2014.351 25541152

[7] Mraz M, Kipps TJ. MicroRNAs and B cell receptor signaling in chronic lymphocytic leukemia. Leuk Lymphoma. 2013; 54:1836–39. 10.3109/10428194.2013.796055 23597135 PMC4144718

[8] Papageorgiou SG, Diamantopoulos MA, Kontos CK, Bouchla A, Vasilatou D, Bazani E, Scorilas A, Pappa V. MicroRNA-92a-3p overexpression in peripheral blood mononuclear cells is an independent predictor of prolonged overall survival of patients with chronic lymphocytic leukemia. Leuk Lymphoma. 2019; 60:658–667. 10.1080/10428194.2018.1461861 29911923

[9] Balatti V, Tomasello L, Rassenti LZ, Veneziano D, Nigita G, Wang HY, Thorson JA, Kipps TJ, Pekarsky Y, Croce CM. *miR-125a* and *miR-34a* expression predicts Richter syndrome in chronic lymphocytic leukemia patients. Blood. 2018; 132:2179–82. 10.1182/blood-2018-04-845115 30242085 PMC6238191

[10] Cerna K, Oppelt J, Chochola V, Musilova K, Seda V, Pavlasova G, Radova L, Arigoni M, Calogero RA, Benes V, Trbusek M, Brychtova Y, Doubek M, et al. MicroRNA miR-34a downregulates FOXP1 during DNA damage response to limit BCR signalling in chronic lymphocytic leukaemia B cells. Leukemia. 2019; 33:403–414. 10.1038/s41375-018-0230-x 30111844

[11] Zhuang Q, Shen J, Chen Z, Zhang M, Fan M, Xue D, Lu H, Xu R, He X, Hou J. MiR-337-3p suppresses the proliferation and metastasis of clear cell renal cell carcinoma cells via modulating Capn4. Cancer Biomark. 2018; 23:515–25. 10.3233/CBM-181645 30452399 PMC13078586

[12] Wang Z, Wang J, Yang Y, Hao B, Wang R, Li Y, Wu Q. Loss of has-miR-337-3p expression is associated with lymph node metastasis of human gastric cancer. J Exp Clin Cancer Res. 2013; 32:76. 10.1186/1756-9966-32-76 24422944 PMC3854519

[13] Zuo XL, Chen ZQ, Wang JF, Wang JG, Liang LH, Cai J. miR-337-3p suppresses the proliferation and invasion of hepatocellular carcinoma cells through targeting JAK2. Am J Cancer Res. 2018; 8:662–74. 29736311 PMC5934556

[14] Demarez C, Gérard C, Cordi S, Poncy A, Achouri Y, Dauguet N, Rosa DA, Gunning PT, Manfroid I, Lemaigre FP. MicroRNA-337-3p controls hepatobiliary gene expression and transcriptional dynamics during hepatic cell differentiation. Hepatology. 2018; 67:313–27. 10.1002/hep.29475 28833283

[15] Piwecka M, Glažar P, Hernandez-Miranda LR, Memczak S, Wolf SA, Rybak-Wolf A, Filipchyk A, Klironomos F, Cerda Jara CA, Fenske P, Trimbuch T, Zywitza V, Plass M, et al. Loss of a mammalian circular RNA locus causes miRNA deregulation and affects brain function. Science. 2017; 357:357. 10.1126/science.aam8526 28798046

[16] Hansen TB, Jensen TI, Clausen BH, Bramsen JB, Finsen B, Damgaard CK, Kjems J. Natural RNA circles function as efficient microRNA sponges. Nature. 2013; 495:384–88. 10.1038/nature11993 23446346

[17] de Thé H, Chomienne C, Lanotte M, Degos L, Dejean A. The t(15;17) translocation of acute promyelocytic leukaemia fuses the retinoic acid receptor alpha gene to a novel transcribed locus. Nature. 1990; 347:558–61. 10.1038/347558a0 2170850

[18] Trotman LC, Alimonti A, Scaglioni PP, Koutcher JA, Cordon-Cardo C, Pandolfi PP. Identification of a tumour suppressor network opposing nuclear Akt function. Nature. 2006; 441:523–27. 10.1038/nature04809 16680151 PMC1976603

[19] Wang ZG, Ruggero D, Ronchetti S, Zhong S, Gaboli M, Rivi R, Pandolfi PP. PML is essential for multiple apoptotic pathways. Nat Genet. 1998; 20:266–72. 10.1038/3073 9806545

[20] Gurrieri C, Capodieci P, Bernardi R, Scaglioni PP, Nafa K, Rush LJ, Verbel DA, Cordon-Cardo C, Pandolfi PP. Loss of the tumor suppressor PML in human cancers of multiple histologic origins. J Natl Cancer Inst. 2004; 96:269–79. 10.1093/jnci/djh043 14970276

[21] Wang K, Long B, Liu F, Wang JX, Liu CY, Zhao B, Zhou LY, Sun T, Wang M, Yu T, Gong Y, Liu J, Dong YH, et al. A circular RNA protects the heart from pathological hypertrophy and heart failure by targeting miR-223. Eur Heart J. 2016; 37:2602–11. 10.1093/eurheartj/ehv713 26802132

[22] Huang Z, Zhang N, Ma W, Dai X, Liu J. MiR-337-3p promotes chondrocytes proliferation and inhibits apoptosis by regulating PTEN/AKT axis in osteoarthritis. Biomed Pharmacother. 2017; 95:1194–200. 10.1016/j.biopha.2017.09.016 28931211

[23] Jost I, Shalamova LA, Gerresheim GK, Niepmann M, Bindereif A. Functional sequestration of microRNA-122 from Hepatitis C Virus by circular RNA sponges. RNA Biol. 2018; 15:1032–1039. 10.1080/15476286.2018.1435248. 29486652 PMC6161685

[24] Zheng Q, Bao C, Guo W, Li S, Chen J, Chen B, Luo Y, Lyu D, Li Y, Shi G, Liang L, Gu J, He X, Huang S. Circular RNA profiling reveals an abundant circHIPK3 that regulates cell growth by sponging multiple miRNAs. Nat Commun. 2016; 7:11215. 10.1038/ncomms11215 27050392 PMC4823868

[25] Memczak S, Jens M, Elefsinioti A, Torti F, Krueger J, Rybak A, Maier L, Mackowiak SD, Gregersen LH, Munschauer M, Loewer A, Ziebold U, Landthaler M, et al. Circular RNAs are a large class of animal RNAs with regulatory potency. Nature. 2013; 495:333–38. 10.1038/nature11928 23446348

[26] Ponente M, Campanini L, Cuttano R, Piunti A, Delledonne GA, Coltella N, Valsecchi R, Villa A, Cavallaro U, Pattini L, Doglioni C, Bernardi R. PML promotes metastasis of triple-negative breast cancer through transcriptional regulation of HIF1A target genes. JCI Insight. 2017; 2:e87380. 10.1172/jci.insight.87380 28239645 PMC5313064

[27] Mazza M, Pelicci PG. Is PML a Tumor Suppressor? Front Oncol. 2013; 3:174. 10.3389/fonc.2013.00174 23847764 PMC3705425

[28] Ito K, Bernardi R, Morotti A, Matsuoka S, Saglio G, Ikeda Y, Rosenblatt J, Avigan DE, Teruya-Feldstein J, Pandolfi PP. PML targeting eradicates quiescent leukaemia-initiating cells. Nature. 2008; 453:1072–78. 10.1038/nature07016 18469801 PMC2712082

