# Supplementary material for: Downregulation of circ_0132266 in chronic lymphocytic leukemia promoted cell viability through miR-337-3p/PML axis
Source: Aging (Albany NY). 2019 Jun 1;11(11):3561–73. doi: 10.18632/aging.101997 (PMC6594798; doi:10.18632/aging.101997)
Supplement: Supplementary Table and Figures [file aging-11-101997-s001.pdf]

SUPPLEMENTARY MATERIALS

Supplementary Table 1. The primers of mRNAs and circRNAs.

RNA	Forward primer	Reverse primer
GAPDH	CTGGGCTACACTGAGCACC	AAGTGGTCGTTGAGGGCAATG
PML	CGCCCTGGATAACGTCTTTTT	CTCGCACTCAAAGCACCAGA
POL	GGTAAAGGAATGGATACGCGAA	CGCACAAGGCGTCAATATCTG
WDR26	CGCCCTGGATAACGTCTTTTT	CTCGCACTCAAAGCACCAGA
hsa_circ_0132266	GTCACTGCGCCCTGATAATG	GGATTACATGACATCTGACCTGA
hsa_circ_0029937	CTGCCTGGTTCTGGGAAAAC	TTTGCTCTGTTCTGGTTCCA
hsa_circ_0004731	AGAAAGTGAGCGAGGAGTCA	CTCCTATTGTCTGCCCTCCTAT

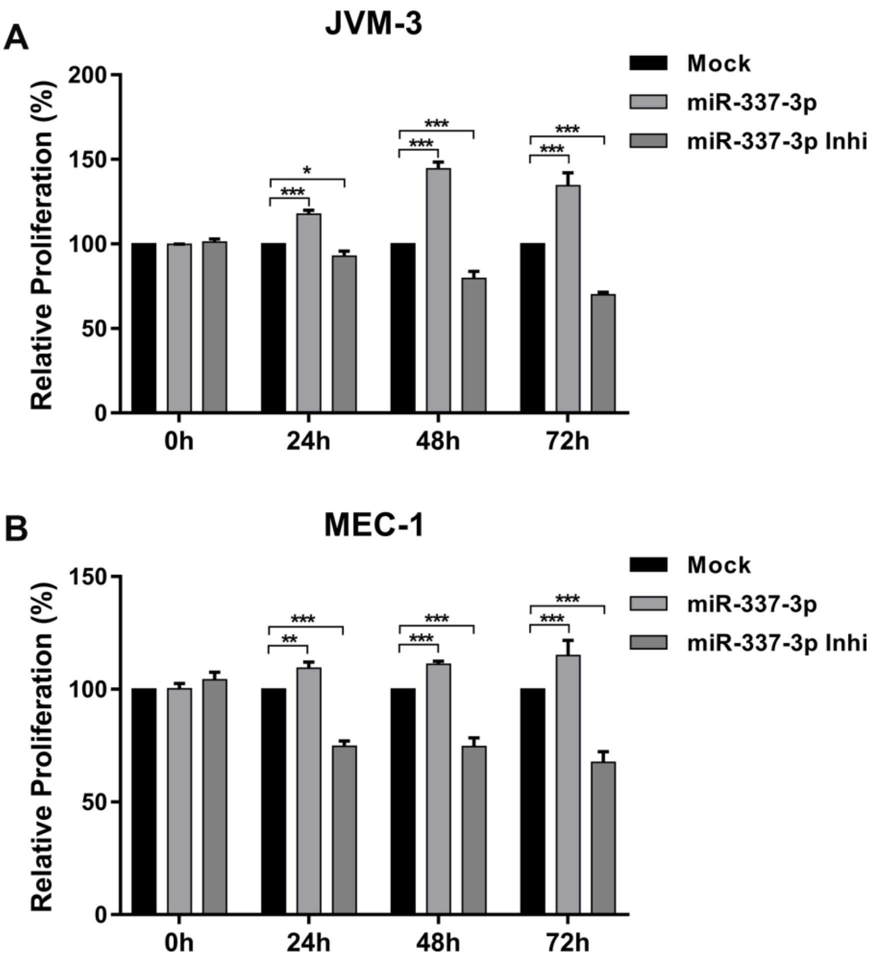

Supplementary Figure 1. miR-337-3p promotes cell proliferation. Relative proliferation of (**A**) JVM-3 and (**B**) MEC-1 after transfecting with miR-337-3p mimics or inhibitor.

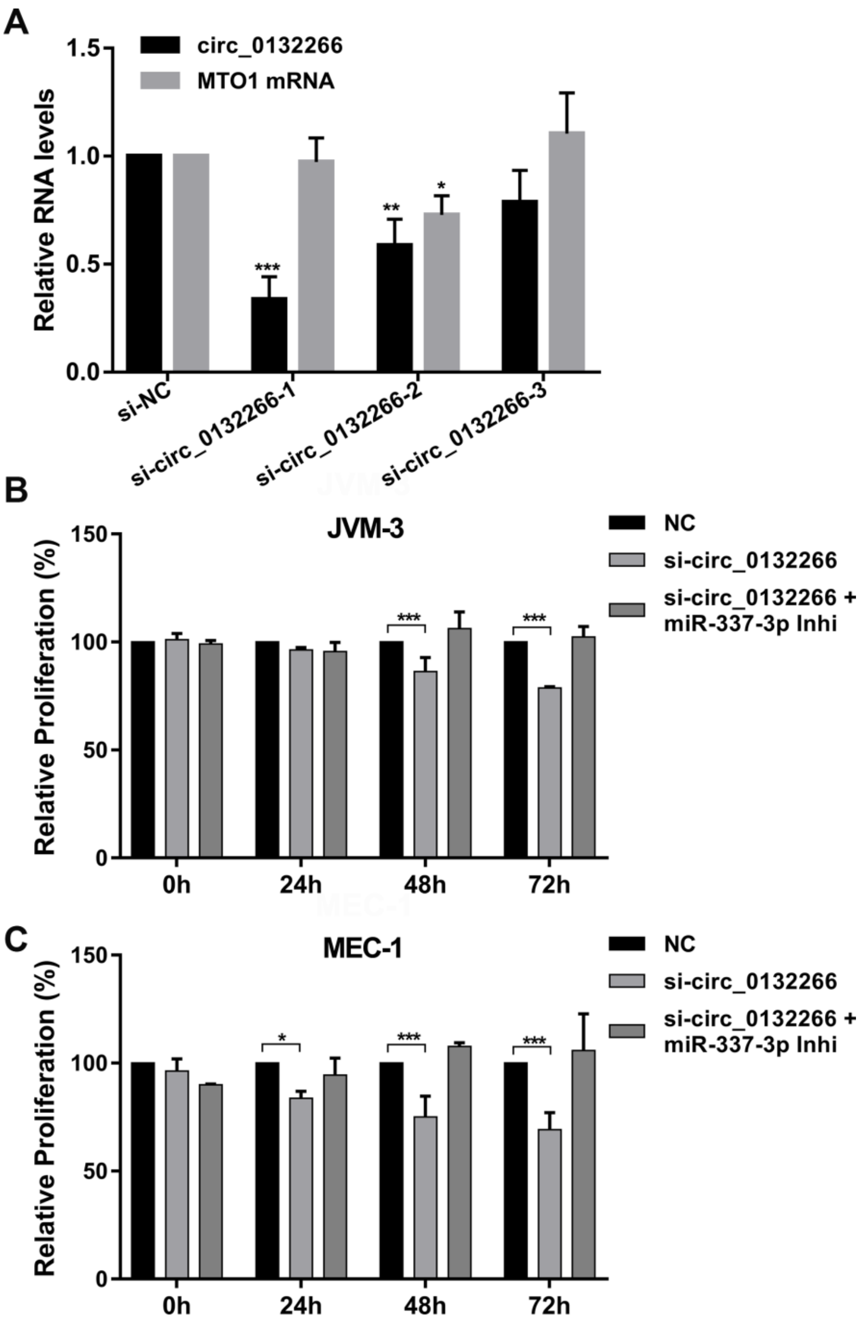

Supplementary Figure 2. circ_0132266 rescues cell proliferation ability of CLL cells induced by miR-337-3p. (**A**) The efficiency of small interfering RNAs knocking down circ_01332266 with specific sequences detected by qRT-PCR. (**B**, **C**) Cell relative proliferation ability detected by CCK8 were calculated.
